# An Imbalanced Generative Adversarial Network-Based Approach for Network Intrusion Detection in an Imbalanced Dataset

**DOI:** 10.3390/s23010550

**Published:** 2023-01-03

**Authors:** Yamarthi Narasimha Rao, Kunda Suresh Babu

**Affiliations:** School of Computer Science and Engineering, VIT-AP University, Amaravathi 522237, Andhra Pradesh, India

**Keywords:** intrusion detection, class imbalance, deep learning algorithms, imbalanced generative adversarial network (IGAN), LeNet 5, LSTM, attacks

## Abstract

In modern networks, a Network Intrusion Detection System (NIDS) is a critical security device for detecting unauthorized activity. The categorization effectiveness for minority classes is limited by the imbalanced class issues connected with the dataset. We propose an Imbalanced Generative Adversarial Network (IGAN) to address the problem of class imbalance by increasing the detection rate of minority classes while maintaining efficiency. To limit the effect of the minimum or maximum value on the overall features, the original data was normalized and one-hot encoded using data preprocessing. To address the issue of the low detection rate of minority attacks caused by the imbalance in the training data, we enrich the minority samples with IGAN. The ensemble of Lenet 5 and Long Short Term Memory (LSTM) is used to classify occurrences that are considered abnormal into various attack categories. The investigational findings demonstrate that the proposed approach outperforms the other deep learning approaches, achieving the best accuracy, precision, recall, TPR, FPR, and F1-score. The findings indicate that IGAN oversampling can enhance the detection rate of minority samples, hence improving overall accuracy. According to the data, the recommended technique valued performance measures far more than alternative approaches. The proposed method is found to achieve above 98% accuracy and classifies various attacks significantly well as compared to other classifiers.

## 1. Introduction

Network intrusion detection faces a growing number of difficulties as the primary method for preventing advanced threat attacks. A long time has passed since the employment of the conventional feature-based IDS [1]. The scope and refresh rate of the established signature database mean that signature-based intrusion detection systems cannot detect all forms of attacks, especially novel attack variations [2]. Researchers have focused significantly on adding new intrusion detection algorithms to address this issue, and one approach is to apply machine learning methods.

Network information security is significantly aided by intrusion detection. However, the network’s traffic types are expanding daily and the features of network behavior are growing more complicated due to the explosive rise of Internet commerce, which poses significant hurdles to intrusion detection [3,4]. The challenge of identifying different harmful network traffic, especially unanticipated hostile network traffic, cannot be ignored. In reality, there are two sorts of network traffic (normal traffic and malicious traffic). Additionally, there are five ways to categorize network traffic: normal, dos, R2L, U2R, and Probe [5,6,7]. Therefore, it is possible to classify ID as a categorization issue. The accuracy of network intrusion detection can be significantly increased by enhancing the classifier’s ability to recognize malicious traffic [8,9,10].

Numerous researchers suggested machine learning (ML) methods to find and identify network attackers, including SVM, KNN, RF, and NB [11,12]. These methods, which have a greater computational cost, are based on conventional ML. They are shallow learners; therefore they do not gain a deeper understanding of their datasets [13]. Additionally, they issue warnings that are partially misleading (i.e., they raise false alarms).

In the past few years, a large number of IDS techniques have been presented based on a variety of approaches, such as mathematical formulations, data mining techniques like machine learning, etc. Poor performances are caused by the difficulty in managing the high-dimensional network traffic data with these statistical formulations and conventional machine learning models [14]. Furthermore, the majority of the techniques used binary classification, such as whether it is an attack or not. Therefore, better approaches are required for IDS, such as deep-learning-based techniques. Due to its powerful learning and feature extraction capabilities, particularly in scenarios involving large datasets, deep learning has been widely recommended for IDS in recent years [15]. Multiple layers are used in deep learning approaches to gradually extract important features from raw input without the need for domain knowledge.

To extract deep features, deep learning offers automated tools. It provides a better data representation to create more advanced models [16,17,18]. Building on recent advances in the field of intrusion detection, recurrent neural networks (RNNs) have emerged as one of the most popular deep learning techniques for categorization and other evaluations of data sequences [19]. Additionally, RNN is a good technique that can show excellent results in subsequent learning and improve anomaly detection in a network system [20].

Therefore, in this paper, we proposed an LSTM-based deep learning model for the multiclass classification of attack detection. To enhance the effectiveness of the classifier, the LeNet 5 and LSTM are hybrid, and IGAN-based class imbalance is implemented. The findings of the experiments are examined and evaluated. The outcomes demonstrate that the NIDS developed in this paper is able to detect intrusions rapidly while maintaining higher accuracy.

The structure of this article is as follows. In the Section 2, we provide a summary of the existing papers. The Section 3 explains the methodology. The result and discussion findings are presented in Section 4, along with an analysis of each experimental outcome. The last section of this article is Section 5, which concludes the paper.

### Novel Contribution

The major key contributions of this research are as follows,

➢ We introduce IGAN, a class imbalance processing method. This strategy can stop random under-sampling from losing crucial samples, as well as the unnecessary time and space costs associated with oversampling. It considerably raises the rate at which minority classes are discovered.
➢ One-hot encoding and normalization operations are performed in preprocessing.
➢ The model accuracy and speed of convergence are both enhanced by data normalization. The class label numeralization of two datasets also employs one-hot encoding.
➢ An ensemble of Lenet 5 and LSTM is employed to classify the various attack categories in NIDS.
➢ The UNSW-NB15 and CICIDS2017 datasets have undergone various experiments. Our proposed network outperforms the state efficiency concerning all other approaches, according to the experimental data.

## 2. Literature Review

*This phase provides a summary of relevant network-based intrusion detection research work.*

Lee. J and Park. K [21] introduced the GAN model to solve the class imbalance problem. GAN is a deep-learning-based unsupervised learning method that produces new virtual data that was comparable to existing data. The GAN can solve the fitting problem along with class overlaps and noise as it resamples by specifying the desired uncommon class. The resampled data is trained by random forest (RF), a common ML technique, to evaluate the classifier’s effectiveness. When compared to existing methodologies, the suggested solution outperforms them.

To address the network intrusion data imbalance problem, Fu et al. [22] suggested an ADASYN oversampling algorithm as the class imbalance approach and a SA model with a higher dropout structure as the data downscaling method. A DL approach for NIDS was proposed for traffic anomaly detection, which integrates a Bi-LSTM network, initially retrieving consecutive characteristics from data traffic via a CNN model, then reconfiguring the weights of every channel via the attention mechanism, and at last employing Bi-LSTM to discover the network of sequential features. When compared to existing techniques, the suggested model achieved 90.73% accuracy and an 89.65% F1 score.

Jiang et al. [23] suggested the ensemble approach that integrates CNN with Bi-LSTM. This method effectively extracts the features of the data. The SMOTE and OSS method was employed to decrease the majority samples and increase the minority samples. OSS was used to decrease the majority samples, and the SMOTE was used to boost the minority samples. In this manner, a balanced dataset for model training is created. The input data is then classified using the network model built by CNN and BiLSTM. Using deep learning’s remarkable characteristics, the algorithm collects characteristics dynamically through recurrent multi-level learning. When evaluated against a testing set, the proposed method produces enhanced outcomes in terms of performance metrics.

Al. S and Denver. M [24] presented an HDL network consisting of CNN and LSTM that is used for better IDS. Furthermore, data imbalance processing, which included the Synthetic SMOTE approach and Tomek-Links sampling approach known as STL, was utilized to mitigate the impacts of class imbalance on system effectiveness. It is possible that you might be interested in the fact that you might be interested in the use of this website. As a consequence, the suggested technique achieved 99.82% accuracy in multi-categorization and 99.16% accuracy in binary categorization. In comparison to existing methods, the suggested approach has obtained relatively good outcomes in identifying network assaults in imbalanced data sets, according to the results.

To tackle the issue of negative and positive instance imbalance in the initial dataset, Cao et al. [25] developed an ensemble sampling method that combines ADASYN and RENN. To solve the issue of feature redundancy, the RF algorithm and Pearson correlation analysis are combined to pick the features. The spatial features are then retrieved using a CNN and further extracted by fusing average pooling and maxpooling, as well as utilizing an attention strategy to apply varying weights to the features, decreasing overhead and boosting method effectiveness. To ensure effective and useful feature learning, the long-distance dependent information features are extracted using a gated recurrent unit (GRU). The experimental results show that the suggested approach yields greater performance.

Mulyanto et al. [26] developed the focal loss NIDS, a cost-sensitive neural network based on focal loss, to tackle the problem of unbalanced data. FL-NIDS was employed in conjunction with DNN and CNN to evaluate intrusion detection data with skewed distributions. To overcome the problem of unbalanced data, focal loss was utilized. When contrasted with other techniques, the presented method applying FL-NIDS in the DNN and CNN framework provides more effectiveness.

Man. J. and Sun. G. [27] presented a NIDS architecture based on DCNN. DCNN with residual blocks was used to learn more essential properties. To detect minor assaults in the testing set, the modified FL function was analyzed instead of the cross-entropy FL to address the imbalanced data issue in the training set. To avoid overfitting, the system is improved with batch normalization and global average pooling. According to test findings, the suggested method can enhance attack detection precision over existing methods. An overview of relevant studies is shown in Table 1.

Despite the high detection accuracies obtained, our related work demonstrated that there are still improvements to be made. Such problems include inconsistent or average accuracy levels and substantial dataset change. The region is still in its early stages of development. The majority of the researchers focused on ML techniques and integrated many algorithms to create a more realistic and effective solution for a detailed dataset with restricted attacks. Many other important classifiers are overlooked in the analysis. Although most of them attempt to address some of the shortcomings of existing oversampling techniques, they are unable to eliminate noise while also distributing the generated samples in a minority of data centers. This is because the closer the samples are to the data center, the higher their contribution to categorization. In addition, the more apparent the traits, the larger the contribution of the samples to categorization. As a consequence, we assume that the approach and work provided in this paper will yield credible findings. This work will enhance and integrate several single learners to train a model more precisely and faster. Greater precision will result in faster training and detection speeds.

## 3. Proposed Methodology

**Figure 1:**
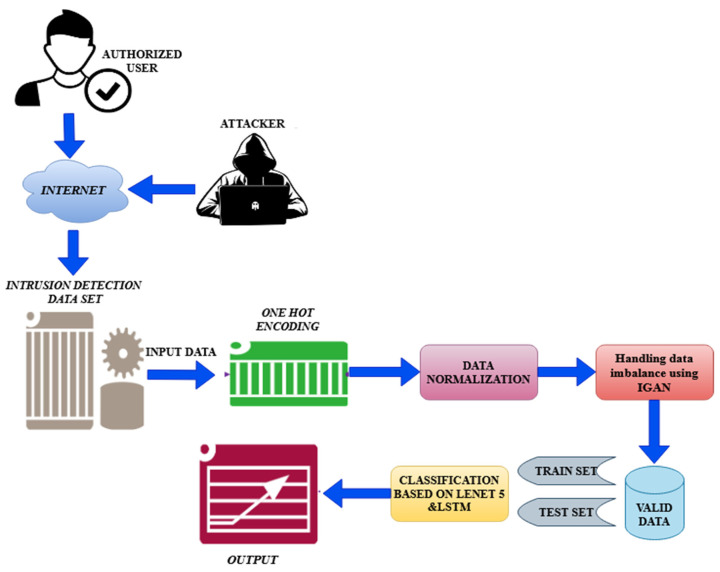
Architecture diagram of the proposed methodology.

The proposed method is thoroughly discussed in this portion. The main framework of the system proposed in this research is given in Figure 1, which is built on the IGAN technique and a hybrid technique of LeNet 5 and LSTM. Data preprocessing is a very important component of data analysis that directly affects prediction accuracy. To make the original data more suitable for the model’s prediction, the data preparation module is in charge of conducting operations on the data, such as one-hot encoding and data normalization.

Resampling the training dataset is the imbalance processing module’s main task to lessen the bias that the original dataset’s imbalance has on the results of experiments. We presented a novel approach called IGAN that integrates over- and under-sampling to produce a perfectly balanced dataset. We provided an ensemble of LeNet 5 and LSTM in the categorization decision module to conclude the attack types. We carry out multi-class categorization employing two different datasets.

### 3.1. Problem Statement

This section discusses the problem statement identified by IDS research using existing methodologies.

An ML-based IDS creates a higher false detection ratio and experiences data imbalance concerns due to a restricted training dataset in the UNSW-NB15 and CICIDS2017 datasets. This imbalance causes problems for classifiers and leads to poor detection accuracy for these minority classes. Existing intrusion detection systems (IDS) are insufficient in dealing with new attack types in networks due to low recognition and detection rates. Overfitting is also another major concern in IDS research. Furthermore, most existing ML-based IDS have a higher computational time. Existing methods are not broadly applicable, because most existing ID systems are incapable of detecting major threats due to out-of-date ID datasets. The data is thought to be noisy, with inaccuracies, and unpredictable, with code or name variances. To alleviate the imbalance issues, we used over-sampling, which involved arbitrarily repeating data in the minority class to boost the minority class’s presence in the sample. Despite the possibility of overfitting, no data was destroyed, and the oversampling method beat the undersampling method.

### 3.2. Preprocessing

We organize the data in such a way that it is ready for the learning algorithm right away. In a circulation manner, the CICDDoS2017 and UNSW-NB15 datasets are provided. Before the component testing, we go through a few processes to collect the data required.

#### 3.2.1. One-Hot Processing

Symbolic features in the dataset are converted into numerical features using the one-hot approach. One-hot encoding is the major often utilized approach for dealing with the numeralization of ordinal attributes since it is a feasible and elegant encoding technique. Ordinal attributes transfer into binary vectors containing one unit with a value of one and the other units are zero. The possibility of feasible numbers corresponding to the category feature is indicated by an entity with several ones.

#### 3.2.2. Normalization

The value of the original data may be excessively high, which could lead to issues like “large numbers to eat decimals”, data processing overflows inconsistent weights, etc. The continuous data is normalized into the range [0, 1] using a conventional scaler. The normalization process removes the measurement unit’s influence from the model’s training and increases the reliance of the training outcome on the properties of the data itself. The min-max method is used to normalize the data. Normalization Equations (1) and (2) present the formula.

(1) $$r^{\prime }=\frac{r-r_{\min}}{r_{\max}-r_{\min}}$$

(2) $$r_{{}_{\max}}=\max\left\{r\right\}$$

Herein, *r*_min_ and *r*_max_ denote minimum and maximum eigenvalues respectively, and the normalized eigenvalue is denoted by *x*′ and the original eigenvalue is denoted by *r*.

### 3.3. Imbalanced Data Handling Using IGAN

The training approach will be more biased towards correctly predicting the majority of samples because the training set’s unbalanced data were used. Therefore, balancing the dataset is very important before classification. In the UNSW-NB15 dataset, ‘Shellcode’, ‘Worms’, ‘analysis’, and ‘backdoor’ classes are increased by the IGAN technique. Similarly, in CICIDS 2017 dataset, the ‘Bot’, ‘MSSQL’, and ‘Heart bleed’ classes are also increased.

A generative model is typically included in a GAN (Generator, *G*) as well as a discriminatory model (Discriminator, *D*). *S* creates noises *z* to create synthesized samples using them as inputs *S*(*z*). D produces the chance *D*(*x*) that sample *x* is the input sample derived from the true distribution. Jensen–Shannon (*JS*) divergence was initially defined as using the following formula to evaluate this similarity:

(3) $$JS(p_{data}∥p_{g})=\frac{1}{2}KL(p_{data∥p_{m}})+\frac{1}{2}KL(p_{g}∥p_{m})\;p_{m}=\frac{1}{2}(p_{data}+p_{g})$$

The maximized data can be represented as

(4) $$\underset{G}{\min}\;\underset{D}{\max}V(D,G)=\underset{G}{\min}\;\underset{D}{\max}(E_{x-p_{data}}(x)[\log D(x)]+E_{z-pz(z)[\log(1-D(G(z)))]}$$

However, the standard GAN is intrinsically incapable of handling class because it seeks to generate samples without taking into account its class unbalance. Additionally, the multilayer perceptron used by the conventional GAN in *G* results in poor expression capabilities. For the minority classes, we, therefore, use an unbalanced data filter in IGAN. This also impacts the generating amount. We go on to think of the lessons as prerequisites for teaching IGAN, including the features of the network. Additionally, we update the design by combining convolutional layers in model *G*, and the expression capability is improved.

#### 3.3.1. System Model

**Figure 2:**
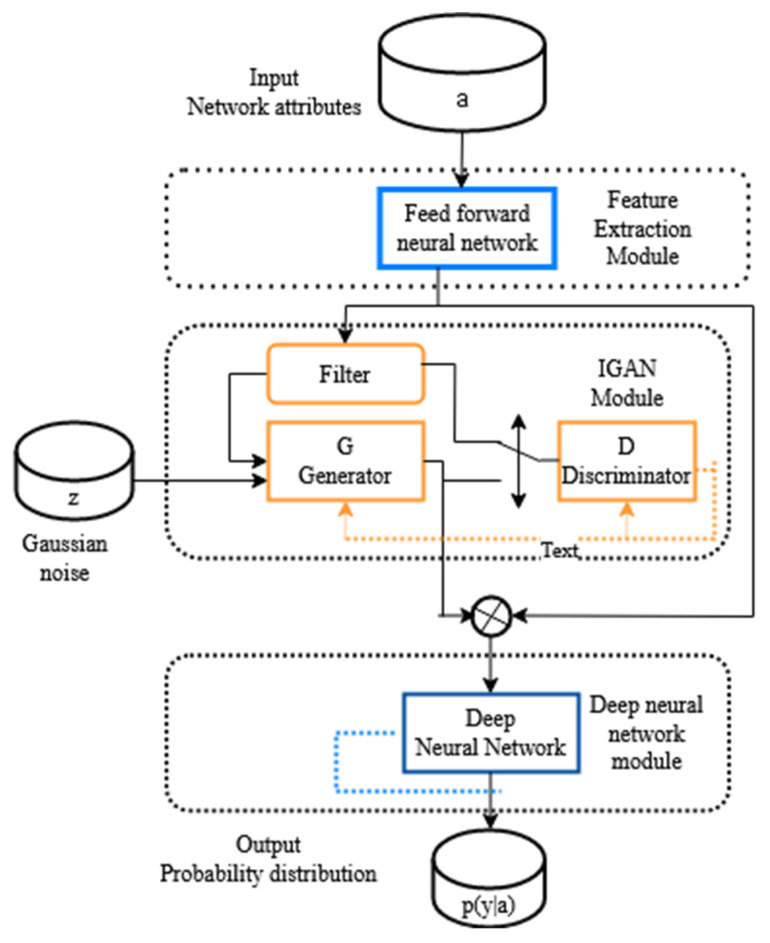
Architecture diagram of IGAN.

IGAN-IDS is made up of three components: extraction of features (FE), IGAN, and DNN. The IGAN-IDS system concept is depicted in Figure 2. To begin, the FE component converts raw network characteristics into latent extracted features. The proposed model then produces instance data, as described in Section 3.3. Lastly, the deep neural network component is trained with balanced samples, and the detection technique is tested on novel data. To recap, IGAN uses network characteristics as input and forecasts their likelihood function *p*(*y*, *j*) across various ID classes.

#### 3.3.2. Model Description

GAN is a technique for learning from unstructured data distributions and generating similar samples. GAN presents two types, one generative and one discriminative. *G* generates a distribution of new instances, whereas *D* differentiates from genuine ones. Following a minimax two-player game among both methods, *G*’s generative distribution may describe the true one, whereas *D* cannot discriminate among the two and converges to 0.5. We proposed the IGAN model. To perform class imbalance ID, we develop an IGAN-based NIDS. To tackle the issue of imbalance in NIDS, we improve the standard generative adversarial network (GAN) by creating examples for minority classes. Discriminator *D*, the unbalanced data filter, and Generator *G* are the three components of IGAN. Minority-class samples are selected for the unbalanced data filter, which then determines the producing quantity. Figure 2 represents the architecture diagram of IGAN.

(a) Imbalanced data filter

It requires ordinary instance *s* = (*x*, *y*) as input and outcome instance $S^{\prime }=(x^{\prime },y^{\prime })$. In common, the filtering process can be stated as follows:

(5) $$s^{\prime }=\left\{s^{\prime }=(x^{\prime },y^{\prime })|s^{\prime }\in s,y^{\prime }\neq \underset{c,\in c}{\arg\max{\;(n}_{c\tau })}\right\}$$

where $c=\left\{c_{1,}........,c_{\tau }\right\}$ the set of various classes $n_{c_{\tau }}$ represents the quality of instances in class $c_{\tau }$. We established a created ratio to objectively reduce the ratio, which represents the split between synthesized and genuine data. *r* = *i*:*j*, while *i* and *j* are the number of synthesized instances. Multilayer perceptron is the main component of the discriminative model *D*.

(b) Discriminator (*D*)

An MLP is used to construct method *D*. *D* is given either generative results $G(z;y^{\prime })$, as well as the corresponding class labels $y^{\prime }$, as input. $y^{\prime }$ must be incorporated in one-hot matrices before the conjunction. *D* calculates the likelihood *D* (*x*; *y*) that a given input (*x*; *y*) is derived from realistic samples rather than *sG*. The following are the connecting layers *d*:

(6) $$d=\max\left(0,\omega_{{}_{d}}v+b_{d}\right)$$

where *v* is the input of every layer, while $\omega_{d}$ and $b_{d}$ are the weights and bias. Convolutional layers and sigmoid output layers, as well as numerous fully linked layers, are used to implement *G*. The linked layers are formalized.

(c) Generator (*G*)

*G* is implemented using various layers. *G* requires minority-class labels $y^{\prime }$ and noises *z* as input and produces relevant features $xG=G(z;y^{\prime })$ for minority classes. In particular, *z* and $y^{\prime }$ can be combined by convolving to $v=[z;y^{\prime }]$. Before summation, $y^{\prime }$ must be integrated into one-hot vectors, which is identical to D.

The convolutional layer performs the following one-dimensional convolution among the kernels *f* and the input *v*:

(7) $$\rho =\left\{\rho =v*f_{e}|\varepsilon \in [1,n_{p}]\right\}$$

As the value function, we use the condensed form of the JS divergence, which will be covered in more detail in the following section.

Data filtering, adversarial learning, and sample production are the three phases of the training phase. When filtering data, as stated in Equation (8), we initially build a subset of minority classes $s^{\prime }$ and then estimate the producing quantity.

In adversarial learning, the outcome samples $s_{G}=(x_{G;y^{\prime }})$. In IDS, the outcome instance $s_{G}$ is combined with the ordinary instance to address the issue of class imbalance. IGAN working process is shown in Algorithm 1. To explain the such process, one possibility is to train on a value $\hat{V}(D;G)$, which is expressed as follows

(8) $$\underset{\theta_{D}}{\max}\hat{V}(D)=\underset{\theta_{D}}{\max}\frac{1}{m}\sum_{i=1}^{m}(\log D(x_{i}^{\prime },y_{i}^{\prime })+\log(1-D(G(z_{i},y_{i}^{\prime }),y_{i}^{\prime })))$$

**Algorithm 1.** IGAN**Input:***s* = (*x*, *y*) the original samples Output: $s_{G}$ The synthesized samples Parameter: *t*, iteration times of *D* per global iteration $s^{\prime }=\left\{s^{\prime }=(x^{\prime },y^{\prime })|s^{\prime }\in s,y^{\prime }\neq \underset{c,\in c}{\arg\max{\;(n}_{c\tau })}\right\}$ While *D* has not converged to 0.5 do For *t* steps do Optimization of the discriminator $\underset{\theta_{D}}{\max}\hat{V}(D)=\underset{\theta_{D}}{\max}\frac{1}{m}\sum_{i=1}^{m}(\log D(x_{i}^{\prime },y_{i}^{\prime })+\log(1-D(G(z_{i},y_{i}^{\prime }),y_{i}^{\prime })))$ End Optimization of the generator Samples can be changed as a batch form End Generating the samples Return $s_{G}$


### 3.4. Classification

**Figure 3:**
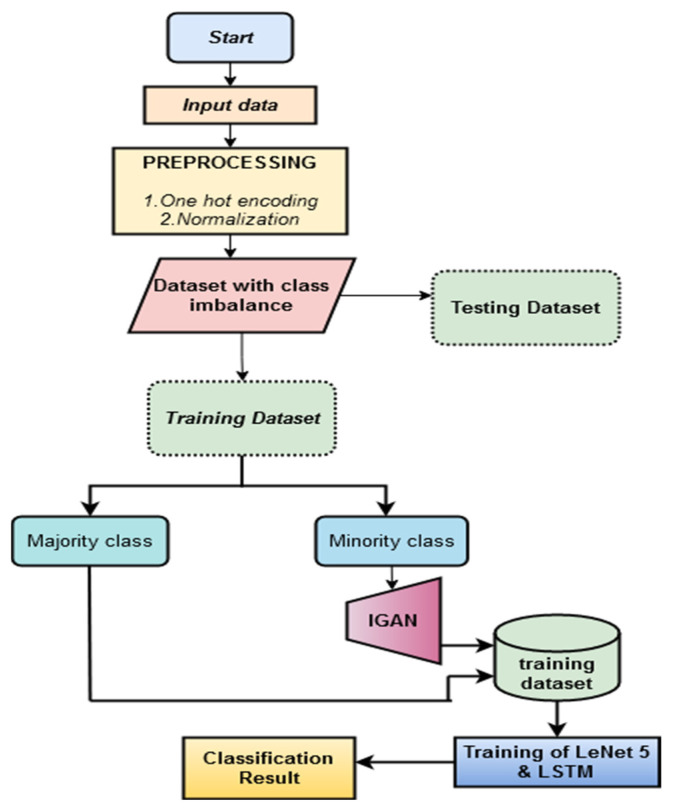
Flowchart of the proposed methodology.

A new classifier LeNet 5 and LSTM are combined for classification to obtain results with higher accuracy and achieve the highest performance of IDS. Figure 3 represents the flow chart of the proposed methodology.

#### 3.4.1. LeNet 5

LeNet-5 was pre-trained as a feature extraction network. It has a total of seven layers. The LeNet-5 precise framework is depicted in Table 2. The LeNet-5’s several weighted layers are built on the idea of reducing blocks of convolutional layers by using shortcut connections. The “bottleneck” blocks are fundamental building blocks that typically adhere to two design principles: employ the same number of filters for extracted features of similar size and twice as many filters for features of half the size. Furthermore, batch normalized is done after every convolution and before activating the ReLU, and down sampling is done with convolution layers with a stride of 2.

Convolutional neural networks are difficult to create because every layer’s input distribution varies during training. This problem can be solved by employing batch normalization (BN) layers, which ensure that the distribution of input data in each layer is stable by normalizing the input to each layer. To increase the speed of model consolidation and training we inserted BN in each convolutional layer. Table 2 shows the architectural details.

Another benefit of our framework is that we used LeakyReLU instead of the standard Tanh, ReLU, and sigmoid activation functions found in the LeNet-5 model. Although it appears to be a linear function, ReLU has a derived function and supports backpropagation, which aids the network in convergent. The ReLU function was chosen to avoid this problem. The equation for the LeakyReLU function

(9) $$f(x)=\{\begin{array}{ll} 0.01x, & \mathrm{for}\;x<0\\ x, & \mathrm{for}\;x\geq 0 \end{array}$$

The function ReLU has as an equation

(10) $$f(x)=\{\begin{array}{c} 0,\;\mathrm{for}\;x<0\\ 1,\;\mathrm{for}\;x\geq 0 \end{array}$$

Our model proposes that in addition to adding the fully connected layer before the output layer and the LeakyReLU activation function after every convolution layer.

#### 3.4.2. Long Short-Term Memory (LSTM)

The LSTM neural network is a commonly used approach for classification tasks. The model includes an LSTM layer and a mean-pooling layer with fully connected input layers. Because it performs better when processing a group of data, we chose LSTM as a great architecture for the classification. We start the method to understand the long-term dependency issue better. In the LSTM neural network, a DL net, long-term dependencies are explicitly designed to be learned. The newly developed gates are designed to store data for a very long time instead. The current input $x_{t}$, the output $c_{t-1}$ of the cells at the (*t* − 1) phase, the terms of bias $b_{g}$, and the time interval of the forget gates are used to determine the $p_{t}$ activation value in forget gates and *t*. Finally, the sigmoid function adjusts all initiate numbers to a scale between 0 and 1

(11) $$p_{t}=sig\left(zy^{x_{t}}+zkk_{t-1}+b_{g}\right)$$

The method handles the specifics and can connect to the cell states in the next section. The value of the initial choice can be computed and included in cell states. Following that, the input activating values are calculated in the subsequent phase.

(12) $$j_{t}=sig\left(Zyx_{t}+Zk_{t-1}+b_{i}\right)$$

Depending on the resolution of the previous two phases, which can be represented by the Hadamard consequence, the following step is a novel cell state. The following equations can be used to represent how memory cells are generated.


(13) $$k_{t}=o_{t}\times \tanh(a_{t})$$

LSTM neurons have a variety of gates, as well as a cell state and a control state. Long-term memory is present in LSTM during the entire sequence.

The weight of the input can be denoted $W_{x}$. The chain rule $W_{x}$ that resulted from the moment *t* is

(14) $$\frac{\partial L_{t}}{\partial W_{t}}=\sum_{k=0}^{t}\frac{\partial L_{t}}{\partial O_{t}}\frac{\partial O_{t}}{\partial S_{t}}\left(\overset{t}{\underset{j=k+1}{∐}}\frac{\partial S_{j}}{\partial S_{j-1}}\right)\frac{\partial S_{k}}{\partial W_{x}}$$

Hence $S_{j}$ represents the unit of RNN in the state at the samples *j*. We stated as for the input layer of tan h in neurons.

(15) $$\prod_{j=c+1}^{t}\frac{\partial S_{j}}{\partial S_{j-1}}=\prod_{j=k+1}^{t}(\tanh W_{s}{)}^{\prime }$$

The range of tan h’s value (.) is 0 to 1. The derivation number of tan h (.) is often reduced throughout the preparatory process, and then t increases.

(16) $$\prod_{j=c+1}^{t}(\tanh W_{s}{)}^{\prime }$$

The controlling gate for the LSTM, as well as information from the previous cell state that is currently secured, was retained.

(17) $$p_{t}=\sigma (Z_{f}.[m_{t-1,}x_{t}]+b_{p})$$

To start the bias and right of the forget gate, the activation function is represented by the symbols, *Wi* and *bi*. The output gate and its input gate can be expressed in a form to determine the current moment:

(18) $$j_{t}=\sigma (Z_{i}.[m_{t-1,}x_{t}]+b_{i})$$

The mathematical model is employed to upgrade the cell state and provide information

(19) $${\tilde{S}}_{t}=\tanh(Z_{S}.[m_{t-1,}x_{t}]+b_{S})$$

(20) $$h_{t}=o_{t}\times \tanh(S_{t})$$

The forget gate is represented as

(21) $$\prod_{j=C+1}^{t}\frac{\partial S_{j}}{\partial S_{j-1}}=\prod_{j=c+1}^{t}(\tanh(\sigma (f_{t}){)}^{\prime }$$

Every gate outcome measure is evaluated, while $b_{\left[i,k,c,o\right]}$ bias vectors

(22) $$o_{t}=sig(Z_{\circ }*[Y_{t-1},X_{t}]+b_{\circ })$$

An appropriate amount of LSTM blocks are combined to form a layer in the empirical technique.

(23) $$Y_{t}=o_{t}•\tanh(S_{t})$$

**Working process of the proposed methodology**

**Step** **1.** Start.
**Step** **2.** Intrusion detection data is an input.
**Step** **3.** Apply preprocessing in the IDS dataset, and one hot-encoding and normalization operation are performed.
**Step** **4.** Symbolic features in the dataset are converted into numerical features using the one-hot approach.
**Step** **5.** Normalization processing removes the measurement unit’s influence from the model training and increases the reliance on the training outcome.
**Step** **6.** Employ an imbalanced generative adversarial network (IGAN) to address the problem of class imbalance by increasing the detection rate of minority classes.
**Step** **7.** Lenet 5 and LSTM are employed to classify the various attack categories in NIDS.
**Step** **8.** End.

## 4. Result and Discussions

To assess the efficiency and effectiveness of the proposed framework, several investigations were carried out on UNSW-NB15 and CICIDS2019 datasets using standard performance metrics. The effectiveness of our proposed approach is then examined. Finally, we evaluate our model’s performance with various start-of-the-art work to confirm the development and feasibility of the proposed model.

### 4.1. Experimental Setting

Using 4 GB RAM and an Intel i5 2.60 GHz processor, it runs Windows 10. The studies were carried out in the Anaconda3 environment using Python and KERAS with Tensor flow as a backdrop. The UNSW-NB15 and CICIDS2017 datasets were utilized for validation in this paper to estimate the effectiveness of our proposed approach. The data samples were split into two sections, one of which was utilized to create a classifier and is referred to as the training dataset. The testing dataset was used in the second step to evaluate the classifier. The parameter configuration is shown in Table 3.

### 4.2. Dataset Description

**UNSW-NB15:** There are nine different attack categories included in the 2.54 million network packets that make up this dataset. This dataset shows severe class imbalances, with total attack traffic making up only 12.65% of the dataset, while regular traffic makes up 87.35% of the overall dataset. Table 4 details the data distribution for every class.

When compared to other sorts of samples, the samples for the categories of backdoors, worms shell code, and analysis are substantially lesser. In particular, worms attacks make up only 130 of the training set’s total attacks and represent 0.07% of it.

**Figure 4:**
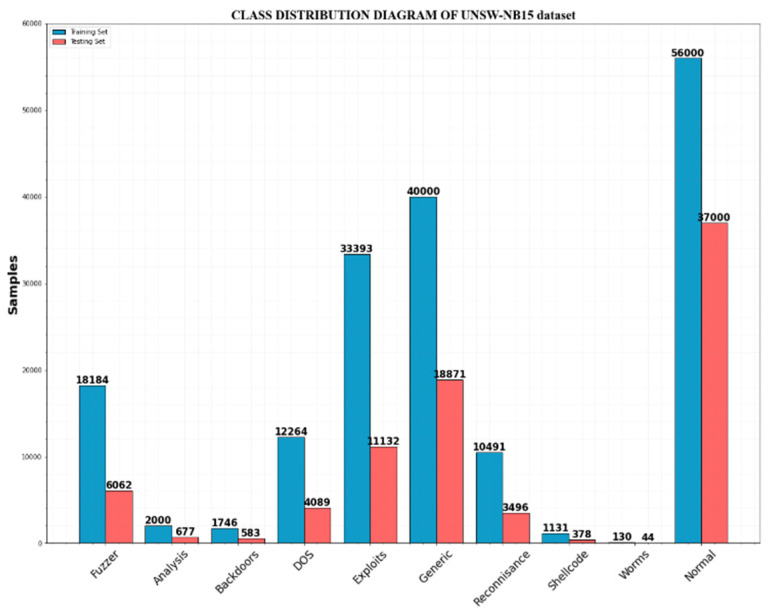
UNSW-NB15 data class distribution.

The ratio of samples of the normal type to samples of the worm attack type across the entire data set is 534:1. Less than 1% of the attack samples for backdoors and shell code are presented. The class distribution of the UNSW-NB15 dataset is shown in Figure 4. Table 5 represents the features of the UNSW-NB15 dataset.

**CICIDS2017 dataset:** There are 2,830,473 samples of network traffic in the dataset, of which benign traffic makes up 80.30% and attack traffic represents 19.70%. There is one normal class and 14 assault types. The assault types include the most prevalent attack types, like port scan, DDoS, web attacks, botnet, DoS, etc. The last column of the dataset, which contains the multiclass label, contains 84 features that were extracted from the generated network traffic. Table 6 provides the data distribution for each class.

The amount of samples from regular traffic is substantially higher than the other categories, and it makes up 80.3% of this data set. DoS Hulk, one of the attack types with the most samples, barely makes up 8.16% of the total data set.

**Figure 5:**
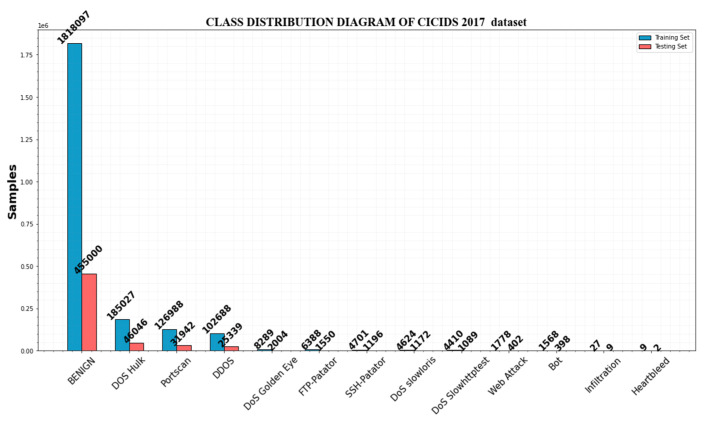
Class distribution of the CICIDS2017 dataset.

The percentages of web attacks, bots, and infiltration in the total data set are 0.08%, 0.07%, and 0.001%, respectively. The lowest amount of samples is for heart bleed. Table 7 and Figure 5 represent the features of the CICIDS2017 dataset.

### 4.3. Evaluation Metrics

The efficiency of the proposed method is evaluated in this research using accuracy (A). In addition to false positive rates (FPR), accuracy, and false positive rates (TPR), recall and precision are also discussed. In the subject of NIDS detection research, these indicators are frequently employed. The calculation formula is presented below. While true positive (*TP*) denotes the Intruder’s proper classification, false positive (*FP*) refers to the misidentification of a legitimate user as an unauthorized user. True negative (*NP*) denotes an accurately classified average user. False negative (*FN*) refers to a situation in which the intruder is mistakenly categorized as a regular user.

The percentage of all correctly classified is measured by accuracy.

(24) $$Accuracy=\frac{TP+TN}{TP+FN+TN+FP}$$

The true positive rate (*TPR*) stands for the proportion of records properly detected over all records with anomalies, which is similar to the detection rate (*DR*).

(25) $$TPR=\frac{TP}{TP+FN}$$

The false positive rate (*FPR*) is the division of wrongly rejected records over all normal records. The following definitions apply to the evaluation metrics:

(26) $$FPR=\frac{FP}{TN+FP}$$

Precision measures the percentage of actual attack records versus expected attack records.

(27) $$precision=\frac{TP}{TP+FP}$$

The percentage of authentic attack samples that were initially detected as attacks in the data set is known as the detection rate (recall rate).

(28) $$recall=\frac{TP}{FN+TP}$$

As a derived effectiveness metric, the F1-score calculates the harmonic mean of precision and recall.

(29) $$F1-Score=\frac{2*precision*recall}{precision+recall}$$

### 4.4. Performance Evaluation of the UNSW-NB15 Dataset

Several investigations on the UNSW-NB15 dataset were done to assess the efficacy of the proposed strategy. Table 8 shows the multi-class categorization results of the proposed technique.

**Figure 6:**
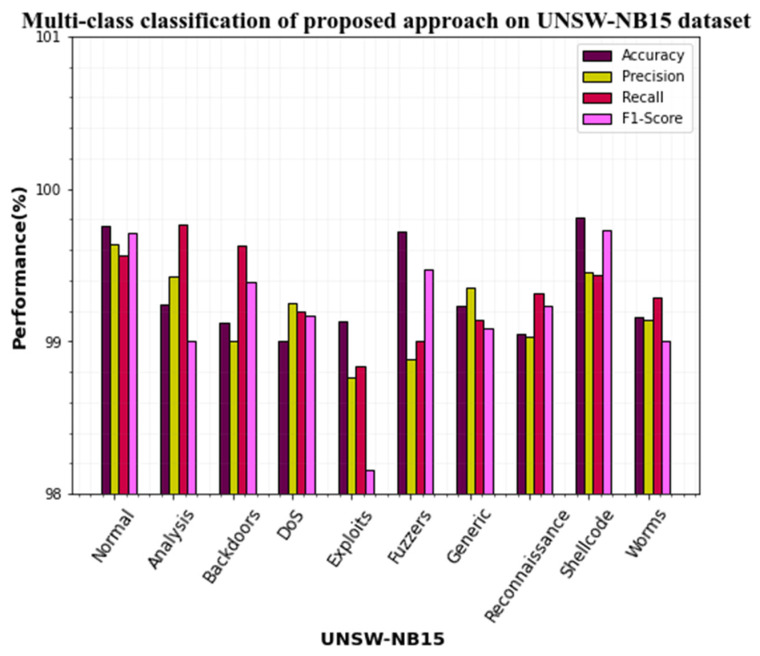
Multiclass classification performances on the UNSW-NB15 dataset.

Table 8 shows that the suggested approach outperforms all other attacks on the CICIDS2019 dataset. All the classes attain above 99% accuracy for all the classes. Specifically, the proposed approach classifies normal, fuzzers, and shellcode with 99.76%, 99.72%, and 99.81% accuracy. Compared to all attacks, the classification performance on the proposed approach of DoS average 99% accuracy. These values are the best. However, compared to all other classes, these values are very low. The graphical representation of this table is represented in Figure 6.

### 4.5. Performance Evaluation on the CICIDS2017 Dataset

Several experiments are carried out using the CICIDS2017 dataset to determine the effectiveness of the suggested approach. The multi-class classification result of our approach is given in Table 9.

**Figure 7:**
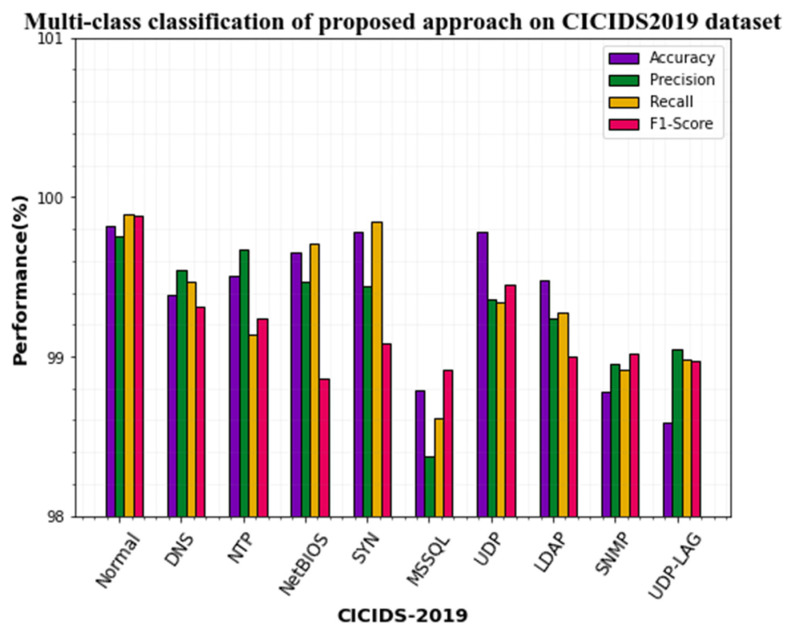
Multiclass classification performances on CICIDS-2019 dataset.

From Table 8, it is observed that the proposed approach’s multi-classification performance is superior and achieves better values for all the attack classes. All the classes attain above 99% accuracy for all the classes. Particularly, the normal, SYN, and UDP classes attain superior results with 99.82%, 99.78%, and 99.78% accuracy, respectively. Moreover, the classification performance on other attack types also provides the best performance. Compared to all the classes, MSSQL detection performance is average with 98.79% accuracy, 98.37% precision, 98.61% recall, and 98.92% f1-score. The graphical representation of Table 8 is shown in Figure 7.

### 4.6. Comparison of CICIDS-2017 and UNSW-NB15 Datasets with Various Approaches

CICIDS-2017 and UNSW-NB15 datasets’ performances can be compared with the existing approaches. The existing approaches like DBN and RBN are compared with the proposed approach.

Table 10 compares the performance of the proposed work to that of other cutting-edge approaches tested on the CICIDS2019 and UNSW-NB15 datasets. Based on the table, the proposed approach-based IDS model achieves the highest results in terms of recall, accuracy, F1-Score, and precision.

**Figure 8:**
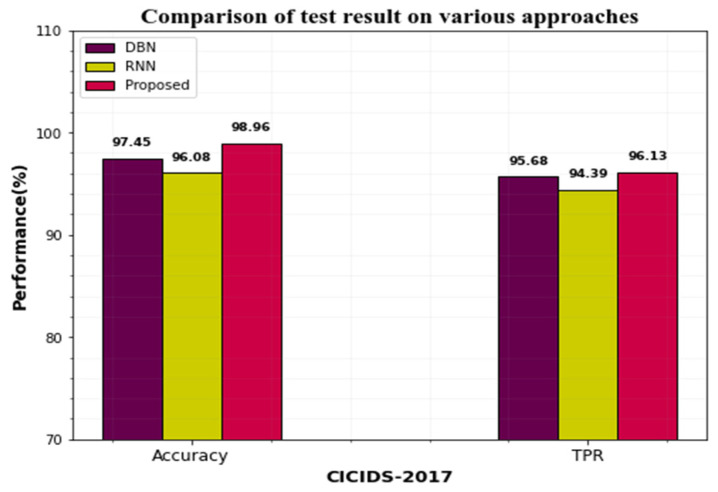
Differentiation performance of the CICIDS dataset proposed approach with existing.

Figure 8 represents the graphical representation of accuracy and TPR on the CICIDS-2017 dataset. When the differentiation can be made with the existing techniques, our proposed approach yields greater performance.

**Figure 9:**
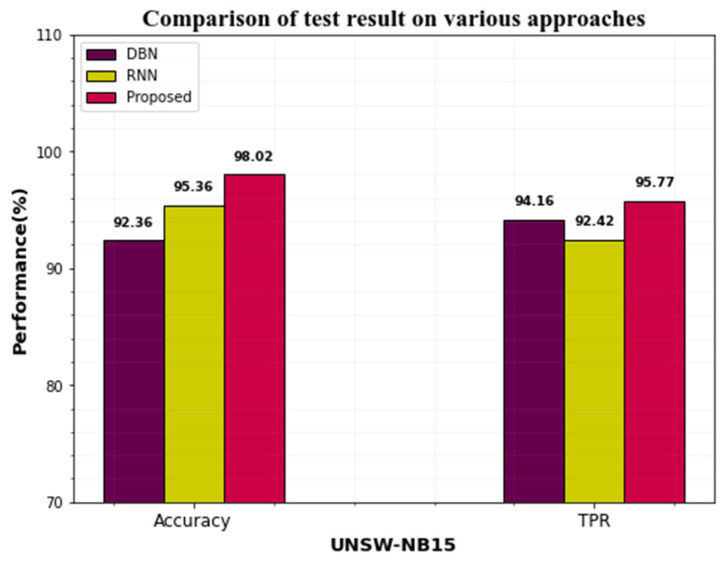
Comparison performance of the UNSW-NB15 dataset proposed approach with the existing one.

Figure 9 depicts the effectiveness of the suggested UNSW-NB15 dataset technique versus the existing one. When compared to existing strategies, our proposed strategy outperforms them.

**Figure 10:**
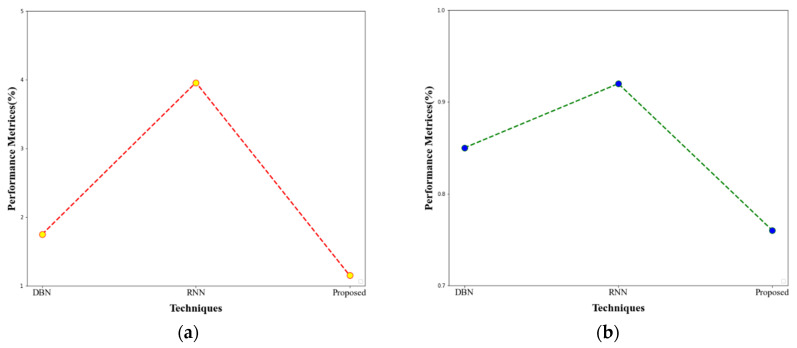
(**a**) FPR comparison for CICIDS-2017 dataset (**b**) FPR comparison for UNSW-NB15 dataset.

When the comparison can be made with the existing techniques, our proposed approach yields greater performance. The FPR metrics performances are compared and represented in Figure 10.

**Figure 11:**
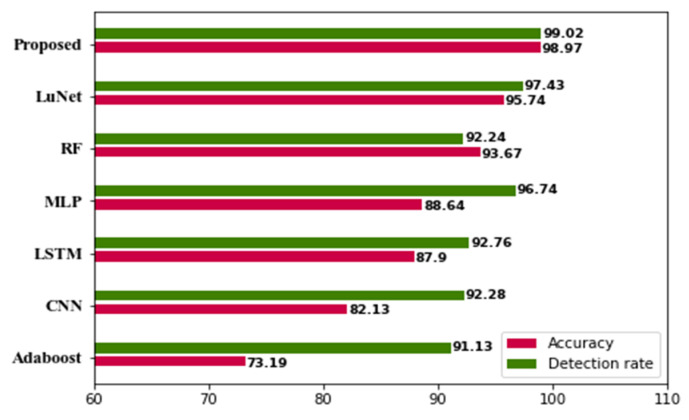
Accuracy and detection rate compared with existing approaches.

**Figure 12:**
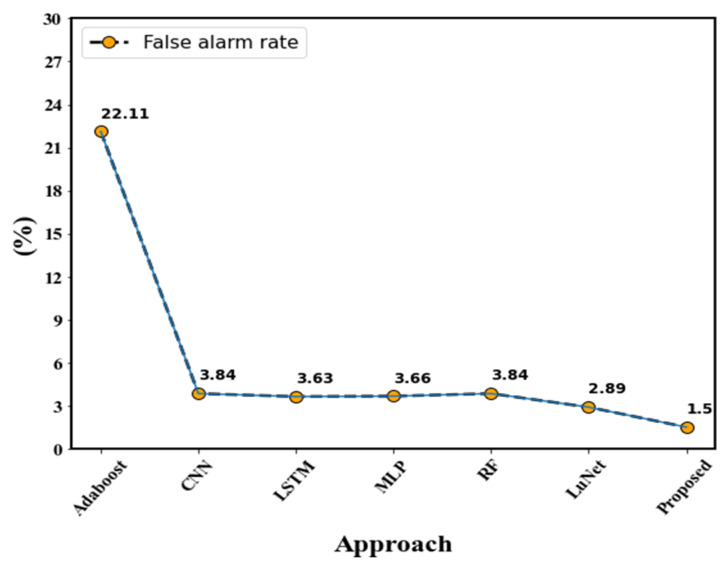
Proposed vs. existing FAR comparison.

Table 11 shows the comparative performance of the proposed approach with the existing one. The existing approaches like Adaboost, CNN, LSTM, MLP, RF, and LuNet are compared. When compared with existing approaches, our proposed approach yields greater performance. Figure 11 and Figure 12 show a comparison of existing approaches with the proposed ones.

The existing approaches, such as LSTM, CNN, RNN-ABC, HCRNNIDS, and ABC- BWO-CONV-LSTM, are differentiated from the proposed method. When differentiating with accuracy metrics, the proposed approach achieves 98.97% accuracy, LSTM achieves 94.73% accuracy, CNN achieves 93.8%, RNN-ABC achieves 96.89%, HCRNNIDS achieves 94.58%, and ABC-BWO-CONV-LSTM achieves 97.03%. While analyzing the performance of accuracy metrics, the proposed method yields the best solution. Then a comparison can be made in precision metrics, and the proposed method yields the best solution, which is 99.06%. The second greater solution presents in ABC-BWO-CONV-LSTM. Similarly, in recall and F1-score, the proposed approach achieves the best outcome. Performance evaluation with the existing approaches is represented in Table 12.

When comparing FPR and FNR metrics performance, the proposed approach achieves a greater solution.

**Figure 13:**
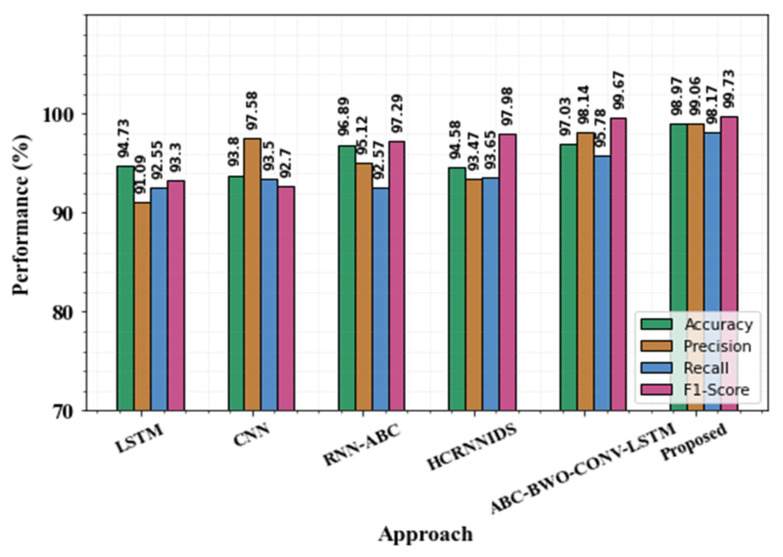
Evaluation of proposed with existing approaches.

**Figure 14:**
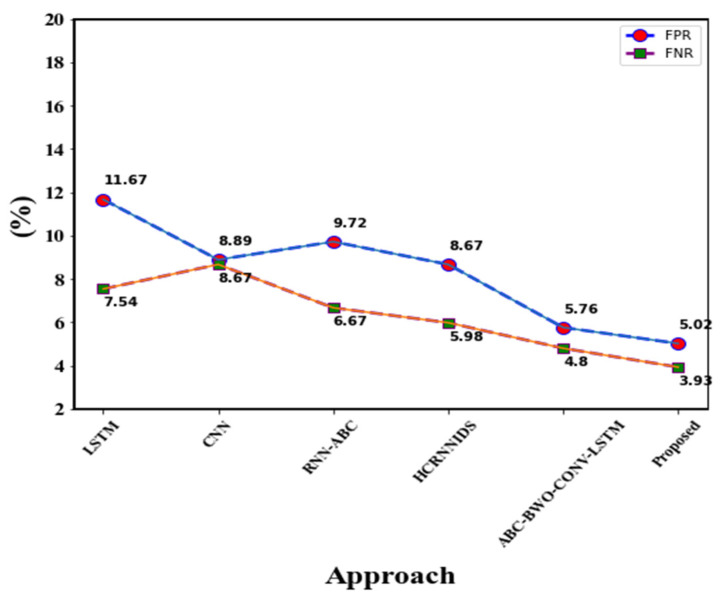
FPR and FNR comparison.

Figure 13 and Figure 14 show the overall performance comparison. Furthermore, a pair-wise t-test was conducted, and the findings confirmed that the proposed technique was statistically significantly distinct from the existing strategies. However, our proposed ensemble model generated a high accuracy of 98.97, a detection rate of 0.989, and a low FNR of 3.93. Additionally, pair-wise t-test statistics of 0.0224 were compared to the existing method.

### 4.7. Data Imbalance Comparison

**Figure 15:**
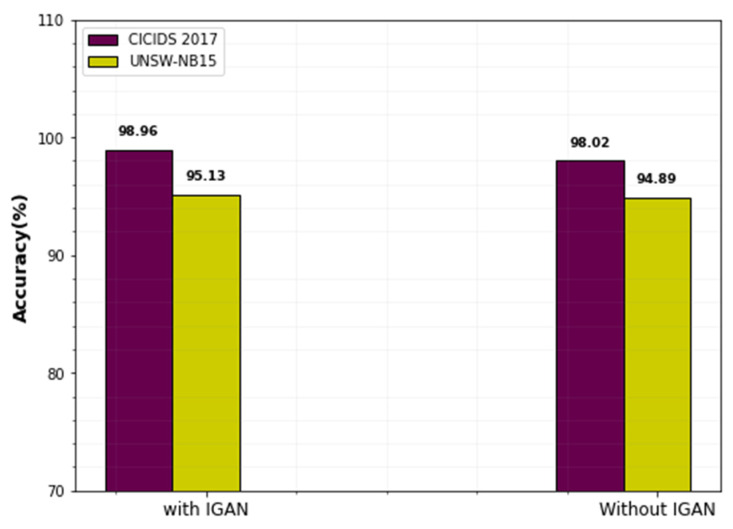
Analysis of data imbalance technique.

The influence of data imbalance on the evaluation criteria of the classifier for both datasets is shown in Figure 15. This graph makes it obvious that using the data augmentation strategy improves classifier performance. In CICIDS2017dataset, the classifier achieves 98.96% accuracy with the IGAN-based imbalance technique. Without IGAN, it achieves only 95.13%. Similarly, the classifier attains 98.02% accuracy for the UNSW-NB15 dataset with the use of data augmentation techniques. The number of training samples generated by this technique is significantly more than training samples without data imbalance.

The same set of training samples is used for every epoch when there is no data augmentation; when there is data imbalance, various sets of training samples are produced for every epoch. As a result, the suggested classifier performs better and achieves higher accuracy in both datasets using the IGAN data imbalance strategy.

### 4.8. Evaluation of Training and Testing Set

**Figure 16:**
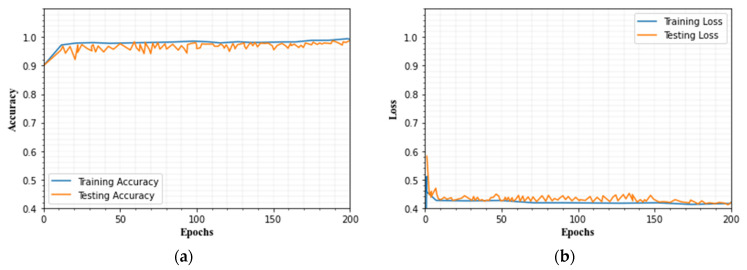
(**a**) Testing and training accuracy, (**b**) testing and training loss for CICIDS2019 dataset.

**Figure 17:**
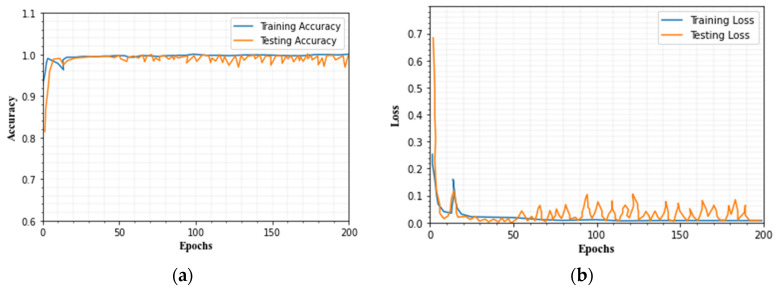
(**a**) Testing and training accuracy, (**b**) testing and training loss for UNSW-NB15 dataset.

Figure 16 and Figure 17 depict a graph of the IDS’s categorization accuracy and loss value as the quantity of iteration steps increases. The graphic shows that the strategy described in this paper has a good convergence impact. We divided the dataset into two stages: training and testing. For this investigation, we generated 75% of the training data and 25% of the testing data. The suggested technique is trained for 200 epochs using the processed training set during the training phase. The learning rate has been set to 0.1.

This portion explores the details of the analytical outcomes employing the proposed approach. Dataset description, preprocessing, handling data imbalance, classification method, and results in the analysis are the five processes in this research. An ensemble of LeNet 5 and LSTM-based approaches are utilized to classify the attack types. In UNSW-NB15 and CICIDS 2017 datasets, some attacks contain minority samples that lead to an imbalance problem that can affect the proposed method’s classification effectiveness. To tackle the class imbalance problem, we introduce the IGAN method. It increases the minority samples.

After that, we classify the attacks with the help of LeNet 5 and LSTM. The batch size, momentum, learning rate, and weight decay are 1, 0.9, 0.03, and 0.001, respectively. The initial learning rate is 0.01. The learning rate in the ReLu layer is saturated. Because the network might be under- or over-fitted, the epoch count is an important training parameter. For this dataset, we trained the network for 200 epochs. The proposed model’s training and testing accuracy vary, and it ranges from 0.98 to 0.99, and loss values range from 0.001 to 0.004.

### 4.9. Case Study

In this example, we show how an attacker can change normal packets so that the NIDS considers their security concerns. As a result, the secured service will reject all ordinary packets, resulting in a huge number of unexpected FPs.

(a) Experimental setup and configuration

For the camera surveillance scenario, we use the UNSW-NB15 and CICIDS2017 datasets, which total 5.37 million packets. In Section 4.1, we take a look at the same setup function. The first half of the packets are employed to train the proposed IGAN method, and the remainder of the packets are utilized to train the Ensemble approach. *T* = 0.04 is chosen to yield the best results.

(b) Results

The main aim of these assaults is to disrupt regular packets to such an extent that the yield of the proposed detection mechanism deviates significantly from distributions, prompting the NIDS to interpret them as malicious packets. To develop adversarial perturbations, the attacker can continue the attack technique on normal data. The extractor component first assesses 10 vectors picked at random from the usual traffic stream. To ascertain which key elements have the most influence on the score value, these properties are intimately connected to the timestamp property. The attacker can then modify the time interval among packets. Drop UDP packets at random with a likelihood of p to achieve this. The sum of the computational gap among two packets is then converted to (1/1 + *p*) t. We take into account the following dropping rates: *p* = {0.1, 0.3, 0.5, and 0.7}. Packets are discarded among index 2 million and 1.8 million. The threshold *T* = 0.04, which yields the best false positive rate and false negative rate under normal conditions. We see that our assault raises the RMSE scores during the packet loss period. While *p* = 0.3, many regular packets with RMSE values more than the threshold are blacklisted as malignant. When the detection threshold is lower, it is simpler to carry such an attack with a lower packet-dropping rate.

## 5. Conclusions

One of the worst issues in all communication networks is NIDS. This study examined the present restrictions and suggested a hybrid IDS approach combining Lenet 5 and LSTM. This article focuses on using the IGAN technique to address the issue of class imbalance in the data set, in contrast to the majority of existing NIDS. Additionally, the hybrid LSTM ensemble model approach is employed to address the issue of complicated models’ extended training and detection times. The outcome of the evaluation using the UNSW-NB15 and CICIDS2017 data sets demonstrates that the proposed NIDS developed in the study has a high detection accuracy, with test sets of 98.96% and 98.02%, respectively. It also achieves the best TPR and FPR in both datasets. In the UNSW-NB15 dataset, the TPR is 95.77% and the FPR is 1.15%. Similarly, in the CICIDS2017 data sets, the TPR is 96.13% and the FPR is 0.76%. The training and detection times of this method are relatively fast when compared to other algorithms. It enhances accuracy and eliminates the FPR and FNR. The proposed approach yield overall 99.06% precision, 98.17% recall, 99.73% F1-Score, and 98.97% accuracy.

Due to the poor error tolerance of IDSs, the UNSW-NB15 data set only comprises a few different types of attacks. In the future, we will integrate additional data sets to cover a wider range of attack types. Last but not least, due to a lack of processing power, testing deeper neural networks with more residual and regular blocks is not possible. As a result, we will carry out more trials in the future with more potent resources and might produce better findings when it comes to identifying network threats. It is planned to integrate various hybrid deep learning techniques in the future and analyze the outcomes. Furthermore, various methods for data balancing will be investigated. Furthermore, the proposed method is intended to be applied instantly to network traffic in a big data environment.

## Figures and Tables

**Table 1:** Summary of existing works.

Reference	Approach	Merits	Demerits
**Lee. J and Park. K [21]**	1D-CNN	The classifier’s effectiveness in predicting minorities has been improved.	In terms of computation, it is more expensive than the other approaches.
**Fu et al. [22]**	Bi-LSTM	Compared to other algorithms, this one is simpler, faster, and less complex.	The quantity of outliers in the data affects performance.
**Jiang et al. [23]**	CNN with Bi-LSTM	The classifiers are one-of-a-kind and low-cost to run.	It is a confusing and time-consuming procedure.
**Al. S and Dener. M [24]**	CNN and LSTM	An algorithm that is simpler, easier, faster, and less difficult.	The main disadvantage of this model is that crucial data can be lost.
**Cao et al. [25]**	RF algorithm	Reduces the rate at which duplicate data is created	In multiclass, the performance standard falls.
**Mulyanto et al. [26]**	FL-NIDS	Faster training speed of undersampling	Only focusing on problems with binary classification
**Man. J and Sun. G [27]**	residual learning	Increased accuracy level	Unable to resolve the issue of imbalance class highly

**Table 2:** Architecture of LeNet 5.

Level	Names of the Layers	Size of Input	Kernel Size	Step Size	Pooled Area	Output Size
**Input**	Input	32 × 32	6 × 6	1		26 × 26
**1st layer**	Convolutional	5@28 × 28		2	2 × 2	6@12 × 12
**2nd layer**	Pool	5@16 × 16	5 × 5	1		16@10 × 10
**3rd layer**	Convolutional	16@14 × 14		2	2 × 2	16@5 × 5
**4th layer**	Pool	16@6 × 6	6 × 6	1		124@2 × 2
**5th layer**	Fully convolutional	1 × 124				1 × 86
**6th layer**	Fully convolutional	1 × 82				1 × 8
**Output**	Output	1 × 7				

**Table 3:** Parameter configuration.

Parameter	Value
**MLP layers**	3
**Epoch**	20
**Batch size**	32
**Decay**	10^−5^
**Momentum**	0.9
**Learning rate**	0.01
**MLP hidden nodes**	48
**RNN hidden units**	128

**Table 4:** UNSW-NB15 data samples of each class.

Class	Training Set Size	Testing Set Size
**Normal**	1,553,132	443,755
**Generic**	150,836	43,097
**Dos**	11,449	3269
**Fuzzers**	16,972	4849
**Backdrops**	1630	466
**Shellcode**	1057	303
**Worms**	122	35
**Exploits**	31,167	8906
**Analysis**	1874	535
**Reconnaissance**	9791	2797

**Table 5:** Features of the UNSW-NB15 dataset.

No	Feature	No	Feature	No	Feature
**1**	rate	16	loss	31	Response_body_ien
**2**	dur	17	synack	32	Ct_src_itm
**3**	dpkts	18	swin	33	ackdat
**4**	Ct_src_dst	19	Sjit	34	State
**5**	dwin	20	Ct_dst_sport_ltm	35	Ct_src_dport_ltm
**6**	spkts	21	smean	36	djit
**7**	proto	22	Ct_flw_http_mthd	37	sbytes
**8**	Ct_dst_src_ltm	23	Ct_srv_src	38	dtrcpb
**9**	Sttl	24	dbytes	39	Is_sm_ips_ports
**10**	Attack_cat	25	dinpkt	40	stcpb
**11**	Is_ftp_login	26	sinpkt	41	Trans_depth
**12**	dttl	27	Ct_state_ttl	42	service
**13**	sloss	28	tcprtt	43	Ct_ftp_cmd
**14**	dload	29	dmean		
**15**	sload	30	Ct_dst_ltm		

**Table 6:** CICIDS2017 data samples of each class.

Class	Training Set Size	Testing Set Size
**BENIGN**	1,591,167	454,620
**Dos Hulk**	161,751	46,215
**Port Scan**	111,251	31,786
**XSS**	457	130
**Infiltration**	26	7
**DDoS**	89,618	25,606
**DoS Golden Eye**	7205	2059
**DoS slow loris**	4057	1159
**FTP**	5516	1588
**Brute force**	1055	301
**SSH**	4128	1179
**DoS slow http test**	3849	1100
**Bot**	1376	393
**MSSQL**	15	4
**Heart bleed**	8	2

**Table 7:** Features of the CICIDS2017 dataset.

No	Feature	No	Feature	No	Feature
**1**	Total Length of Bwd Packets	25	Forward IAT Std	49	Bwd IAT Max
**2**	Init_Win_bytes_forward	26	Bwd Packets Length Max	50	Forward Packets Length Min
**3**	protocol	27	Forward Packets Length Std	51	Fwd IAT Max
**4**	Subflow Forward Packets	28	Backward IAT Total	52	Fwd IAT Min
**5**	Forward Header Length	29	Forward Header Length1	53	Average Bwd Segment Size Fwd Packets/s
**6**	ECE Flag Counts	30	Backward IAT Std	54	Bwd IAT Mean
**7**	Subflow Fwd Bytes	31	Total backward packets	55	Flow duration
**8**	Source port	32	Backward IAT Min	56	Total Length of forwarding Packets
**9**	Subflow Bwd Bytes	33	Forward PSH Flags	57	Subflow Bwd Packets
**10**	Down/Up Ratio	34	Total fwd packets	58	Fwd Packet Length Max
**11**	Average Packet Size	35	Bwd Header Length	59	Destination port
**12**	Avg Fwd Segment Size	36	Forward Packet Length Mean	60	RST Flag Count
**13**	Label	37	SYN Flag Counts	61	Forward IAT mean
**14**	Idle Min	38	Active Mean	62	Backward Packet Length Std
**15**	Packet Length Variance	39	PSH Flag Counts	63	Min Packet Length
**16**	min_seg_size_fwd	40	Flow IAT Std	64	Flow IAT Max
**17**	FIN Flag Counts	41	Backward Packet Length Mean	65	Active Max
**18**	URG Flag Counts	42	Packet Length Std	66	Max Packet Length
**19**	Acknowledge Flag Count	43	Flow Bytes/s	67	Flow Packets/s
**20**	Packet Length Mean	44	Backward Packets/s	68	Active Min
**21**	Active Std	45	Idle Mean	69	Forward IAT Total
**22**	Backward Packet Length Min	46	Idle Max	70	Flow IAT Min
**23**	Init_Win_bytes_bwd	47	Flow IAT Mean		
**24**	act_data_pkt_fwd	48	Idle Std		

**Table 8:** Multi-class classification of the proposed approach on the UNSW-NB15 dataset.

Attack Types	Precision	F1-Score	Accuracy	Recall
**Normal**	99.64	99.71	99.76	99.56
**Analysis**	99.43	99	99.24	99.77
**Backdoors**	99	99.39	99.12	99.63
**DoS**	99.25	99.17	99	99.20
**Exploits**	98.76	98.16	99.13	98.84
**Fuzzers**	98.88	99.47	99.72	99
**Generic**	99.35	99.09	99.23	99.14
**Reconnaissance**	99.03	99.23	99.05	99.32
**Shellcode**	99.45	99.73	99.81	99.44
**Worms**	99.14	99	99.16	99.29

**Table 9:** Multi-class classification of the proposed approach on the CICIDS2019 dataset.

Attack Types	Precision	Accuracy	F1-Score	Recall
**Normal**	99.75	99.82	99.88	99.89
**DNS**	99.54	99.39	99.31	99.47
**NTP**	99.67	99.51	99.24	99.14
**NetBIOS**	99.47	99.65	98.86	99.71
**SYN**	99.44	99.78	99.08	99.85
**MSSQL**	98.37	98.79	98.92	98.61
**UDP**	99.36	99.78	99.45	99.34
**LDAP**	99.24	99.48	99	99.28
**SNMP**	98.95	98.78	99.02	98.92
**UDP-LAG**	99.05	98.59	98.97	98.98

**Table 10:** Comparison of the test results with various approaches.

Dataset	Approaches	Accuracy	TPR	FPR
**CICIDS-2017**	DBN	97.45	95.68	0.85
	RNN	96.08	94.39	0.92
	Proposed	98.96	96.13	0.76
**UNSW-NB15**	DBN	92.36	94.16	1.75
	RNN	95.36	92.42	3.96
	Proposed	98.02	95.77	1.15

**Table 11:** Differentiation of the proposed approach with various existing approaches.

Approaches	Accuracy	Detection Rate	False Alarm Rate
**Adaboost**	73.19%	91.13%	22.11%
**CNN**	82.13%	92.28%	3.84%
**LSTM**	87.90%	92.76%	3.63%
**MLP**	88.64%	96.74%	3.66%
**RF**	93.67%	92.24%	3.84%
**LuNet**	95.74%	97.43%	2.89%
**Proposed**	98.97%	99.02%	1.50%

**Table 12:** Performance evaluation with existing approaches.

Approaches	Accuracy (%)	Precision (%)	Recall (%)	F1-Score (%)	FPR (%)	FNR (%)
**LSTM**	94.73	91.09	92.55	93.3	11.67	7.54
**CNN**	93.8	97.58	93.5	92.7	8.89	8.67
**RNN-ABC**	96.89	95.12	92.57	97.29	9.72	6.67
**HCRNNIDS**	94.58	93.47	93.65	97.98	8.67	5.98
**ABC-BWO-CONV-LSTM**	97.03	98.14	95.78	99.67	5.76	4.8
**Proposed**	98.97	99.06	98.17	99.73	5.02	3.93

## Data Availability

The publicly available data set can be found at: https://www.kaggle.com/datasets/mrwellsdavid/unsw-b15 accessed on 3 August 2015, https://www.kaggle.com/datasets/vinesmsuic/cicids2017aniddataset?select=pro-Monday-0.5v2.csv accessed on 25 June 2016.
